# The Recent Medical Legislation

**Published:** 1880-09

**Authors:** 


### Editorial.

### THE RECENT MEDICAL LEGISLATION.

Considerable interest and inquiry have been excited in professional circles in regard to the Act, passed by the last legislature of this state, regulating the licensing of physicians and surgeons. On general principles, we have regarded the enactment of laws to be salutory, by means of which the wide-spread impositions practiced upon the credulous public in matters pertaining to the cure of disease, could be controlled, or at least restricted. The efforts which have been made heretofore in this direction, have signally failed to accomplish the desired object, and the conviction has been forced upon the profession that we must depend upon the increasing intelligence of the community for relief from the various forms of quackery which prevail extensively in every portion of our country. Through what influence we are not informed, another effort was made in the last session of the legislature, to solve this vexed question, with results, we predict quite as unsatisfactory as that obtained from all previous legislation upon the subject.

With a view to obtain correct notions of the scope and purpose of the present law, we give a brief resumé of each section:

Section 1 provides that a person shall not practice physic and surgery within the State, unless he is twenty-one years of age.

Section 2 provides that "every person now *lawfully* engaged in the practice of physic and surgery, shall, on or before the first day of October, 1880, etc., etc., register in the County Clerk's Office his name, residence, place of birth," etc. etc.

Section 3 provides that "a person who violates either of the two preceding sections or, who shall practice physic or surgery under cover of a diploma illegally obtained, shall be deemed to be guilty of a misdemeanor," etc.

Section 4 requires "a person with a diploma conferring upon him the degree of Doctor of Medicine, issued by an incorporated university, medical college, or medical school *without the state*, to exhibit the same to the faculty of some incorporated medical college or medical school *of this state* with satisfactory evidence of good moral character, and if said diploma and qualifications are approved, they shall endorse said diploma, for which he shall pay the sum of twenty dollars to the Dean of said faculty," etc., etc.

Section 5 provides that "the degree of Doctor of Medicine, lawfully conferred by any incorporated medical college or university of this state, shall be a license to practice after being duly recorded and verified by oath as required by section two."

Section 6 provides that "nothing in this act shall apply to the Army, Navy, or Hospital Marine Medical Corps, nor to any person who has practiced medicine or surgery *for ten years last past*," *etc.*

The law plainly requires every person, whether he has graduated from a medical school within the state or without the state, and who has been engaged in practice under ten years, to register at the County Clerk's Office before Oct. 2, 1880, but it does not apply to those who have been practicing over ten years.

Section six exonerates all the latter class, whether they possess a legal parchment, or a purchased diploma, or none at all from the requirements of this act.

What has been gained by the enactment of this law for the protection of the medical profession or the public, it would be idle to conjecture. The various forms of imposture will flourish as before, as far as any law is concerned, and the profession stands before the public in the future as in the past, upon its merits, depending for protection and success upon the scientific merits and achievements of its members, and upon the high and noble principles and purposes, which have always inspired it.

In this connection we are permitted to state that the faculty of the Buffalo Medical College have decided, in order to enable such of the profession who have to comply with section 4 of this law, to endorse the diplomas of all members in good standing of the Erie Co. Medical Society, free of charge. It will be seen that the college early and generously appreciates the important relations it holds to the profession, and places the power the law imposes at its service.

